# A novel dosimetric metrics-based risk model to predict local recurrence in nasopharyngeal carcinoma patients treated with intensity-modulated radiation therapy

**DOI:** 10.1186/s13014-021-01911-5

**Published:** 2021-09-23

**Authors:** Wenjun Liao, Jinlan He, Zijian Liu, Maolang Tian, Jiangping Yang, Jiaqi Han, Jianghong Xiao

**Affiliations:** Department of Radiation Oncology, West China Hospital, Sichuan University, Chengdu, 610041 China

**Keywords:** Nasopharyngeal carcinoma, Intensity-modulated radiation therapy, Local recurrence, Dosimetric metrics, Dose volume histogram

## Abstract

**Background:**

To develop a risk model based on dosimetric metrics to predict local recurrence in nasopharyngeal carcinoma (NPC) patients treated with intensive modulated radiation therapy (IMRT).

**Methods:**

493 consecutive patients were included, among whom 44 were with local recurrence. One-to-two propensity score matching (PSM) was used to balance variables between recurrent and non-recurrent groups. Dosimetric metrics were extracted, and critical dosimetric predictors of local recurrence were identified by Cox regression model. Moreover, recurrent sites and patterns were examined by transferring the recurrent tumor to the pretreatment planning computed tomography.

**Results:**

After PSM, 44 recurrent and 88 non-recurrent patients were used for dosimetric analysis. The univariate analysis showed that eight dosimetric metrics and homogeneity index were significantly associated with local recurrence. The risk model integrating *D*_5_ and *D*_95_ achieved a C-index of 0.706 for predicting 3-year local recurrence free survival (LRFS). By grouping patients using median value of risk score, patients with risk score ˃ 0.885 had significantly lower 3-year LRFS (66.2% vs. 86.4%, *p* = 0.023). As for recurrent features, the proportion of relapse in nasopharynx cavity, clivus, and pterygopalatine fossa was 61.4%, 52.3%, and 40.9%, respectively; and in field, marginal, and outside field recurrence constituted 68.2%, 20.5% and 11.3% of total recurrence, respectively.

**Conclusions:**

The current study developed a novel risk model that could effectively predict the LRFS in NPC patients. Additionally, nasopharynx cavity, clivus, and pterygopalatine fossa were common recurrent sites and in field recurrence remained the major failure pattern of NPC in the IMRT era.

**Supplementary Information:**

The online version contains supplementary material available at 10.1186/s13014-021-01911-5.

## Background

Nasopharyngeal carcinoma (NPC), originating from the mucous epithelium of the nasopharynx, is a heterogeneous malignancy highly prevalent in South China and Southeast Asia [1, 2]. Due to the concealed location and high radiosensitivity of NPC, radiotherapy has been the most effective treatment modality for NPC [1, 3]. Although excellent local control has been achieved with the wide use of intensity-modulated radiation therapy (IMRT), local recurrence remains an important cause of treatment failure in approximately 10% of advanced NPC [4–6]. What’s worse, for patients with locally recurrent NPC, the salvage treatment options are limited and the prognosis is miserable, with 5-year overall survival ranging from 28 to 60% in patients with rT3-T4 disease [6–8].

There is no doubt that the probability of local control is highly correlated with dosimetric metrics in an IMRT planning. Therefore, some metrics have been recommended to evaluate the feasibility of an IMRT planning [9]. Previous studies have investigated how dosimetric metrics influenced the local control rate of NPC patients [10, 11]. However, most of the studies did not consider enough significant metrics in dose-volume histogram (DVH), nor did them exclude the effect of other clinical confounding factors, such as treatment modalities, on the prognosis of NPC. Therefore, reliable dosimetric metrics for IMRT planning evaluation remain scanty, and the association between dosimetric metrics and local recurrence has not yet been well-established. It is important to find out the most relevant dosimetric metrics to describe a dose rationality and to predict local recurrence of NPC patients in routine practice.

In addition, due to the technical advantages of IMRT, NPC treated with IMRT had its unique recurrent characteristics when compared with that treated with two-dimensional or three-dimensional conformal radiotherapy [12, 13]. Although previous studies have examined local failure patterns of NPC treated with IMRT and indicated that local recurrence mainly occurred in high dose area [14, 15], it is critical to examine the recurrent sites and patterns with a larger cohort, which would contribute to gain insight into recurrent features, target contouring, and planning optimization of NPC in the IMRT era.

Therefore, the present study aimed at analyzing the effect of dosimetric metrics on local recurrence of NPC patients, and subsequently developing a predictive risk model for local recurrence free survival (LRFS) of patients. Moreover, the recurrent sites and patterns of NPC treated with IMRT were also elucidated.

## Methods

### Study population

**Fig. 1:**
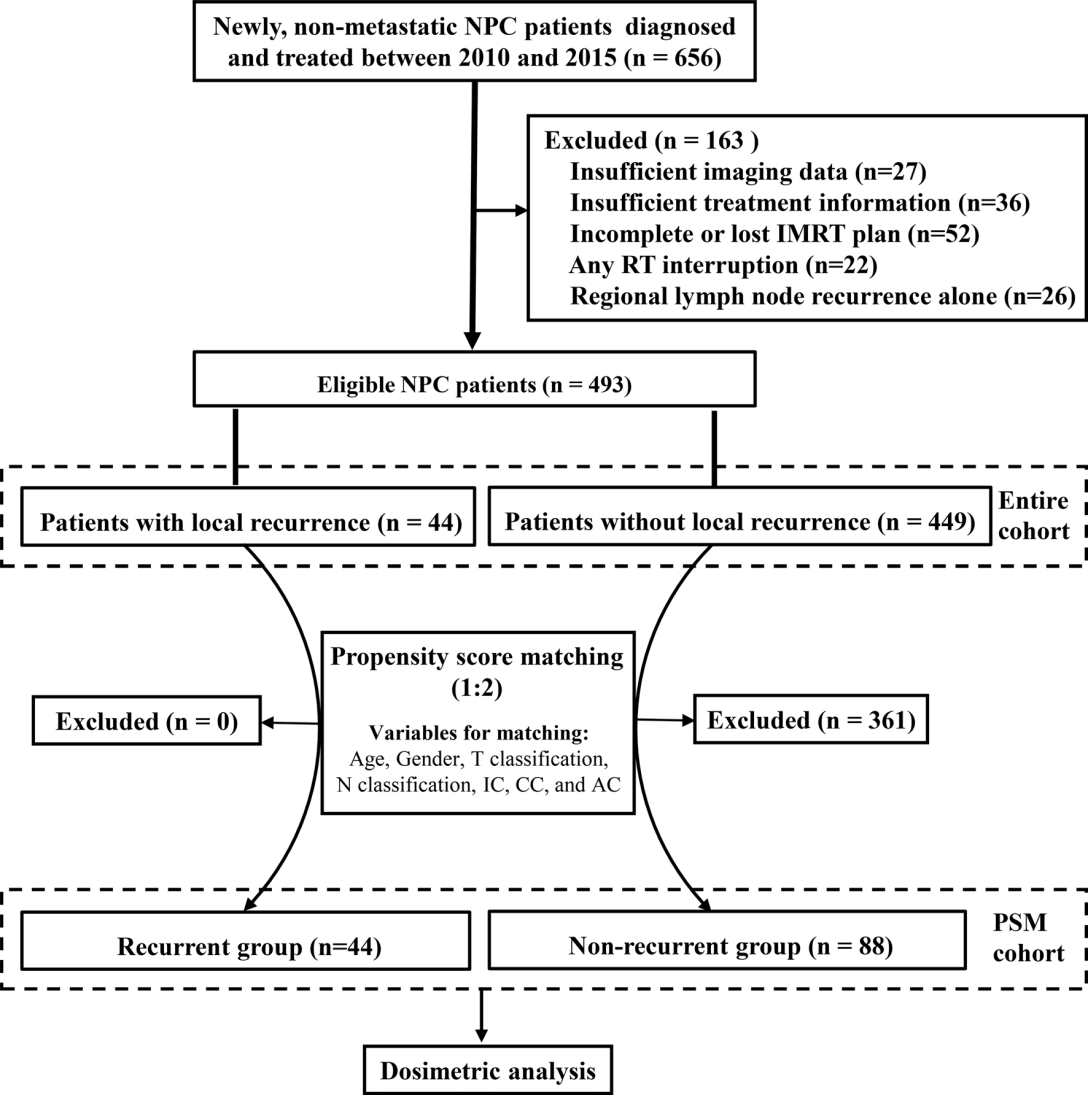
The flowchart of patient selection. NPC, nasopharyngeal carcinoma; IMRT, intensity-modulated radiation therapy; RT, radiotherapy; IC, induction chemotherapy; CC, concurrent chemotherapy; AC, adjuvant chemotherapy; PSM, propensity score matching

Newly diagnosed NPC patients treated by curative-intent chemo-radiotherapy in West China Hospital between January 2010 and December 2015 were reviewed. The eligible criteria were as follows: histologically confirmed NPC; no distant metastasis at initial diagnosis; achieved complete remission (CR) after initial treatment. The main exclusion criteria included regional lymph node recurrence alone, history of other malignancy or insufficient treatment or image data. The flowchart of patient selection was illustrated in Fig. 1.

All patients were restaged according to the eighth edition of the American Joint Committee on Cancer (AJCC). This study was approved by the Ethics Committee on Biomedical Research of the hospital and the informed consent was waived.

### Target definition and delineation

Target volumes were delineated according to the International Commission on Radiation Units and Measurements (ICRU) reports 83 [16] and the treatment protocol of our cancer center. The nasopharynx gross tumor volume (GTVnx) and node gross tumor volume (GTVnd) were determined by physical, endoscopic, and imaging examinations. The positive retropharyngeal lymph nodes were delineated together with the GTVnx. For patients receiving induction chemotherapy (IC), the primary tumor volume before IC was utilized for GTVnx delineation, and the volume of lymph nodes after IC was utilized for GTVnd delineation. High-risk clinical target volume (CTV1) was defined as the GTVnx plus a 5–10 mm margin and the whole nasopharynx mucosa. Low-risk clinical target volume (CTV2) was defined as CTV1 plus a 5–10 mm margin, which included the posterior part of nasopharyngeal cavity, posterior third part of maxillary sinus, posterior ethmoid sinus, the inferior part of sphenoid sinus and cavernous sinus, skull base, the anterior third part of clivus and cervical vertebra, parapharyngeal space, and pterygopalatine fossa. The above margins were initially obtained with automatic 3D expansion, and then slightly adjusted manually according to tumor characteristics. The clinical target volume for bilateral lymphatic drainage area (CTVnd) routinely included levels II to V nodal regions. The planning tumor volumes (PTVs) were created by adding 2–3 mm margin with automatic 3D expansion to the above target volumes.

In this study, two types of prescribed radiation doses were used for PGTVnx based on patients’ clinical stage. For patients with T4 classification or with bulky primary tumor, a dose of 74 Gy in 33 fractions at 2.24 Gy/fraction was administrated to the PGTVnx. For the other patients, a dose of 70 Gy in 33 fractions at 2.12 Gy/fraction was administrated. All patients received 70 Gy in 33 fractions to PGTVnd, 60 Gy in 33 fractions to PCTV1, and 56 Gy in 33 fractions to the PCTV2 and PCTVnd.

The planning goal was to deliver at least 95% of prescription dose to 100% of the PTVs without exceeding the dose tolerance of organs at risk (OARs). We mainly followed the protocol of Radiation Therapy Oncology Group (RTOG) trial 0225[17] and the protocol from a published study [14]. In short, the ideal maximal point dose should be less than 54 Gy for brainstem, optic chiasma and optic nerve, 45 Gy for spinal cord, and 65 Gy for temporal lobe. However, if these constraints could not be fulfilled, acceptable criteria were to allow less than 60 Gy to 1% volume for brainstem, optic chiasma and optic nerve, and less than 50 Gy to 1 cc for spinal cord, and less than 70 Gy maximal point dose for temporal lobe.

IMRT was delivered with 6 MV X-ray beams modulated using either a step-and-shoot IMRT or a rotational technique (volumetric modulated arc therapy, VMAT). In addition, the technique of simultaneous integrated boost (SIB) was adopted in our center.

### Chemotherapy

In terms of patients with T1-2 and N0, radiotherapy alone was adopted, and the other patients (T3-4/N +) were treated with radiotherapy combined with cisplatin-based chemotherapy. IC, concurrent chemotherapy (CC), and adjuvant chemotherapy (AC) were included in this study. The common IC and AC protocols included PF (cisplatin 80 mg/m^2^ d1-3 + 5-fluorouracil 800 mg/m^2^/day/ d1-5), TPF (docetaxel 60 mg/m^2^ d1 + cisplatin 60–80 mg/m^2^ d1-3 + fluorouracil 800 mg/m^2^/day/ d1-5), GP (gemcitabine 1000 mg/m^2^ d1 + cisplatin 80 mg/m^2^ d1-3), and TP (docetaxel 80 mg/m^2^ d1 + cisplatin 80 mg/m^2^ d1-3). CC consisted of cisplatin (80 mg/m^2^ d1-3) was given every three weeks during the period of radiotherapy.

### Follow-up

Patients were evaluated every week during radiotherapy including physical and hematological examinations. After treatment, follow-ups were regularly scheduled every three months in the first two years, thereafter, every six months until death or loss to follow-up (the last follow-up was on Dec 31, 2019). Follow-up included physical examinations, nasopharyngoscopy, magnetic resonance imaging (MRI) for the head and neck, computed tomography (CT) for the chest, ultrasonic/ CT/ MRI of the abdomen, and whole-body bone scan if necessary. Local recurrence referred to the disappearance of the primary tumor after radical treatment but the occurrence of new lesion six months later, and local recurrence free survival (LRFS) was defined as the duration from the date of diagnosis to the date of local recurrence. No patient was lost to follow up in this study, and the median LRFS of these 493 patients was 58.4 months (Range, 7.6 to 100.6 months).

### Propensity score matching

Propensity score matching (PSM) [18] was used to filter out clinical variables affecting tumor prognosis between patients with or without local recurrence so that the baseline characteristics of the two groups were comparable. Variables entering the PSM model included age, gender, T classification, N classification, IC, CC, and AC. In this study, one to two matching was performed (Fig. 1).

### Dosimetric metrics extraction and definition of failure patterns

In this study, we mainly focused on the recurrence of nasopharynx tumor (local recurrence). *D*_x_ was defined as the minimum absorbed dose that covers x% of the volume of the target. Dosimetric metrics of the PGTVnx from *D*_5_ to *D*_95_ in steps of 5 were calculated and extracted from the pretreatment DVH through in-house script run by RayStation (Raysearch laboratories, Sweden) treatment planning system. In addition, *D*_1_, *D*_2_, *D*_98_, *D*_99_, *D*_ave_ (the average dose of the target), *D*_min_ (the minimum dose of the target), and *D*_max_ (the maximum dose of the target) were also extracted. Homogeneity index (HI^#^) was defined as *D*_5_/*D*_95_ according to the report of AAPM Task Group 101 (TG 101) [19], and HI^*^ was defined as (*D*_2_-*D*_98_)/*D*_50_ based on the ICRU 83 [16].

For patients with local recurrence, first the MRI images obtained at the time of local recurrence were transferred to the RayStation. The pretreatment planning CT served as the basis for registration, namely that the MRI images were moved to be registered with the CT images. Bony, vascular, and muscular structures adjacent to the failure were utilized to guide the co-registration process, which was repeated until satisfactory visual agreement was acquired between the MRI and CT images. Then, the recurrent tumor volume (RTV) was delineated on MRI images, and copied from MRI images onto the pretreatment planning CT. Finally, the exact site and extent of each tumor were compared with the pretreatment planning CT, concentrating on the 95% isodose lines. Doses received by RTV was calculated and analyzed with DVH. The patterns of failure were classified into in field failure (95% of RTV was within the 95% isodose), marginal failure (20% to 95% of RTV was within the 95% isodose), and outside field failure (less than 20% of RTV was inside the 95% isodose) [20].

### Statistical analysis

Statistics analysis was performed using SPSS software package (Version 22.0, IBM SPSS Inc) and R software package (Version 3.5). Categorical variables were compared by Pearson chi-square test. In univariate analysis, log-rank test was performed for category variables, such as age, gender, T classification, N classification, IC, CC, and AC, and a Cox regression model was used for continuous variables, such as dosimetric metrics. For those factors with *p* < 0.05 in univariate analysis, a multivariate Cox regression analysis using a stepwise method with likelihood-ratio was performed to identify key dosimetric metrics and develop model for local recurrence, which was completed by “survival” package and “survminer” package. Survival analysis was calculated by Kaplan–Meier method, and survival curves of different groups were compared by log-rank test. A two-tailed *p-*value < 0.05 was considered significant.

## Results

### Patient characteristics

A total of 493 NPC patients were included in this study. Clinical characteristics and treatment modalities were summarized in Table 1. In detail, 44 patients had local recurrence and 449 patients did not. Before matching, the proportion of patients who received CC in the recurrent group was significantly lower than that of patients in the non-recurrent group (*p* = 0.042). To exclusively analyze the effect of dosimetric metrics on tumor recurrence, PSM was used to balance clinical variables which might affect tumor control, and a new cohort, the PSM cohort, was constructed. The new cohort included 44 recurrent patients and 88 non-recurrent patients, thereby eliminating the differences of observed baseline variables (*p* > 0.05) (Table 1).

**Table 1:** Clinical characteristics of patients with and without recurrence

Variable	Entire cohort		*P *value	PSM cohort		*P *value
	Recurrent (n = 44)	Non-recurrent (n = 449)		Recurrent (n = 44)	Non-recurrent (n = 88)	
Age			0.068			0.705
≤ 46	16 (36.4)	228 (50.8)		16 (36.4)	35 (39.8)	
> 46	28 (63.6)	221 (49.2)		28 (63.6)	53 (60.2)	
Gender			0.382			0.432
Male	34 (77.3)	319 (71.0)		34 (77.3)	73 (82.9)	
Female	10 (22.7)	130 (29.0)		10 (22.7)	15 (17.1)	
T classification			0.593			0.255
T1-2	14 (31.8)	161 (35.9)		14 (31.8)	37 (42.0)	
T3-4	30 (68.2)	288 (64.1)		30 (68.2)	51 (58.0)	
N classification			0.822			0.803
N0-1	19 (43.2)	186 (40.5)		19 (43.2)	36 (40.9)	
N2-3	25 (56.8)	263 (58.5)		25 (56.8)	52 (59.1)	
IC			0.272^*^			0.550^*^
Yes	38 (86.4)	410 (91.3)		38 (86.4)	80 (90.9)	
No	6 (13.6)	39 (8.7)		6 (13.6)	8 (9.1)	
CC			0.042			0.170
Yes	32 (72.7)	380 (84.6)		32 (72.7)	73 (82.9)	
No	12 (27.3)	69 (15.4)		12 (27.3)	15 (17.1)	
AC			0.246			0.368
Yes	18 (40.9)	145 (32.3)		18 (40.9)	29 (33.0)	
No	26 (59.1)	304 (67.7)		26 (59.1)	59 (67.0)	
Prescription						0.119
70 Gy	25 (56.8)	312 (69.5)	0.085	25 (56.8)	62 (70.5)	
74 Gy	19 (43.2)	137 (30.5)		19 (43.2)	26 (29.5)	
RT technique			0.485			0.318
IMRT	21 (47.7)	239 (53.2)		21 (47.7)	34 (38.6)	
VMAT	23 (52.3)	210 (46.8)		23 (52.3)	54 (61.4)	

IC, induction chemotherapy; CC, concurrent chemotherapy; AC, adjuvant chemotherapy; RT, radiotherapy; IMRT, intensity-modulated radiation therapy; VMAT, volumetric modulated arc therapy

*P* values were calculated by χ^2^ test or Fisher’ exact test (^*^)

### Feature selection and prediction model developing

**Fig. 2:**
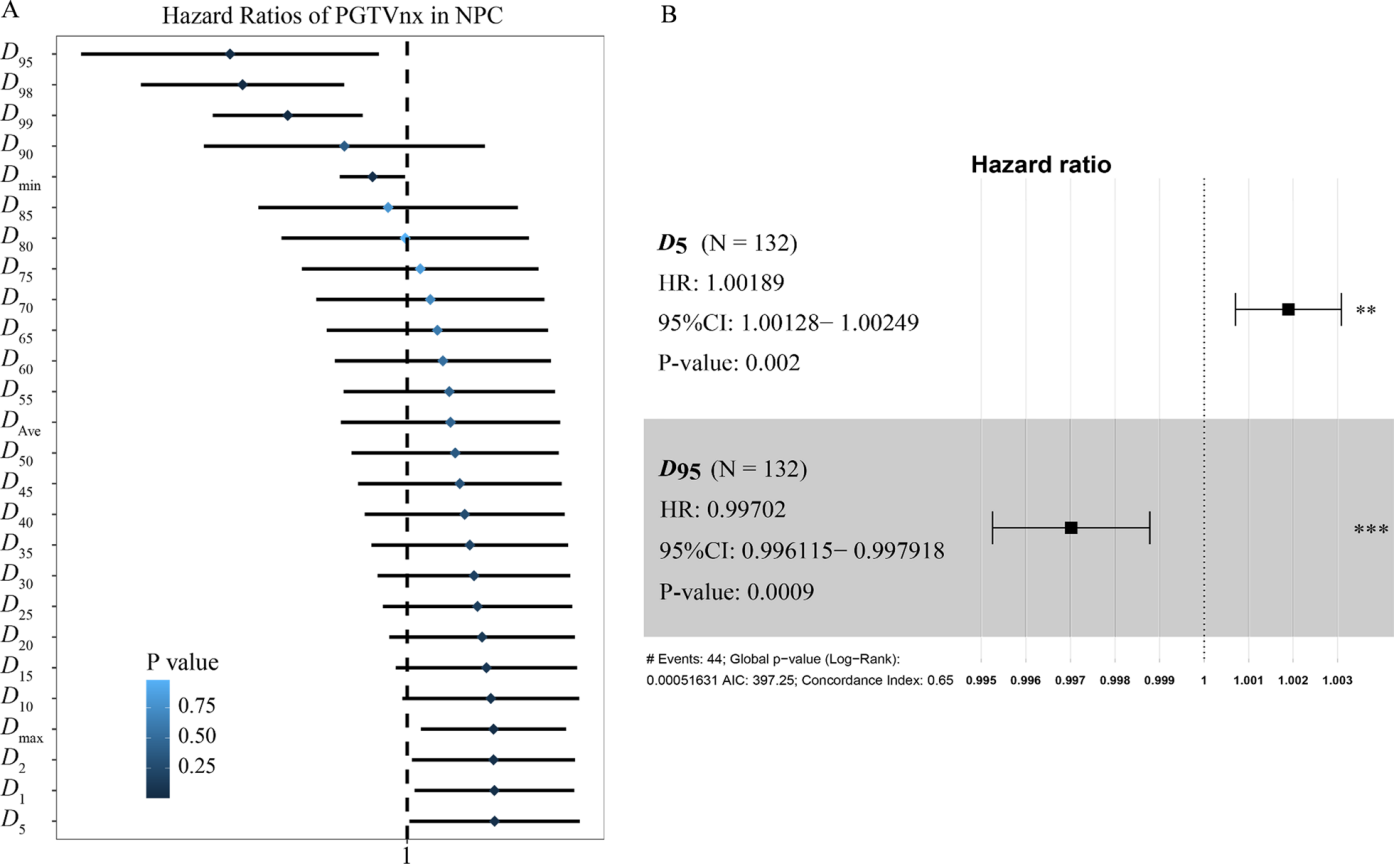
Univariate and multivariate analysis of dosimetric metrics. **a** Eight dosimetric metrics were significantly correlated with LRFS derived from the univariate analysis using a cox regression model; **b** Two dosimetric metrics were statistically correlated with LRFS derived from the Cox regression analysis using a stepwise method with likelihood-ratio. LRFS, local recurrence free survival

Table 2 shows comparison of dosimetric metrics between recurrent and non-recurrent patients. Significant differences were found between the two groups in metrics of *D*_max_, *D*_1_, *D*_2_, *D*_95_, *D*_98_, and *D*_99_ (all *p* < 0.05). And, *D*_5_ and *D*_min_ were close to be significant (*p* = 0.057 and *p* = 0.073, respectively). Subsequently, a univariate analysis including clinical factors and all dosimetric metrics in the PSM cohort was conducted. The results showed that none of the clinical factors was significantly associated with local recurrence (Table 3). However, eight dosimetric metrics including *D*_max_, *D*_1_, *D*_2_, *D*_5_, *D*_95_, *D*_98_, *D*_99_, and *D*_min_ were significantly associated with local recurrence (Fig. 2a and Additional file 1: Table S1), among which *D*_95_, *D*_98_, *D*_99_, and *D*_min_ were protective factors. To identify the critical dosimetric metrics that mostly affected local relapse of patients, the Cox regression model was performed on the eight statistically significant variables derived from the univariate analysis. The result showed that only *D*_5_ (*p* = 0.002) and *D*_95_ (*p* < 0.001) were independent factors for predicting local recurrence (Fig. 2b and Additional file 2: Table S2). A predictive model was then constructed according to the coefficient of the two dosimetric metrics acquired from the Cox regression analysis, and the risk score was calculated as follows: Risk score = *D*_5_ * 0.0019- *D*_95_ * 0.0030.

**Table 2:** Comparison of dosimetric metrics between recurrent and non-recurrent patients

Metrics	Recurrent group (Gy) (n = 44)	Non-recurrent group (Gy) (n = 88)	*P *value
*D*_max_	79.72 ± 3.22	78.47 ± 2.80	0.024
*D*_1_	78.60 ± 2.83	77.58 ± 2.56	0.039
*D*_2_	78.22 ± 2.78	77.25 ± 2.50	0.047
*D*_5_	77.65 ± 2.61	76.77 ± 2.41	0.057
*D*_10_	77.11 ± 2.48	76.34 ± 2.33	0.083
*D*_15_	76.74 ± 2.38	76.06 ± 2.30	0.112
*D*_20_	76.42 ± 2.30	75.81 ± 2.24	0.147
*D*_25_	76.14 ± 2.24	75.60 ± 2.20	0.187
*D*_30_	75.88 ± 2.18	75.39 ± 2.17	0.224
*D*_35_	75.63 ± 2.13	75.20 ± 2.13	0.271
*D*_40_	75.39 ± 2.10	75.01 ± 2.10	0.331
*D*_45_	75.14 ± 2.06	74.82 ± 2.07	0.394
*D*_50_	74.90 ± 2.01	74.62 ± 2.05	0.459
*D*_55_	74.65 ± 1.99	74.42 ± 2.01	0.541
*D*_60_	74.38 ± 1.96	74.20 ± 1.98	0.636
*D*_65_	74.10 ± 1.91	73.97 ± 1.95	0.720
*D*_70_	73.79 ± 1.86	73.71 ± 1.90	0.826
*D*_75_	73.43 ± 1.80	73.41 ± 1.85	0.965
*D*_80_	73.00 ± 1.73	73.07 ± 1.80	0.842
*D*_85_	72.47 ± 1.65	72.61 ± 1.75	0.665
*D*_90_	71.66 ± 1.57	71.96 ± 1.71	0.325
*D*_95_	70.04 ± 1.66	70.76 ± 1.71	0.022
*D*_98_	67.77 ± 2.40	68.89 ± 1.93	0.005
*D*_99_	66.14 ± 3.11	67.52 ± 2.40	0.006
*D*_min_	56.66 ± 7.47	58.71 ± 5.38	0.073
*D*_ave_	74.53 ± 1.87	74.30 ± 1.95	0.519

*D*_x_ was defined as the minimum absorbed dose that covers x% of the volume of the target. *D*_ave_ represented the average dose of the target. *D*_min_ represented the minimum dose of the target. *D*_max_ represented the maximum dose of the target

*P* values were calculated by t test; Data was denoted as mean ± standard deviation

**Table 3:** Univariate analysis of LRFS by log-rank test according to clinical factors

Variable	HR	95% CI	*P *value
Age (≤ 46 vs ˃ 46)	1.083	0.586–2.002	0.799
Gender (Male vs Female)	0.920	0.527–1.739	0.816
T classification (T3-4 vs T1-2)	1.477	0.783–2.786	0.229
N classification (N2-3 vs N0-1)	0.958	0.527–1.739	0.887
IC (Yes vs No)	0.801	0.315–2.038	0.641
CC (Yes vs No)	0.637	0.328–1.237	0.183
AC (Yes vs No)	1.350	0.740–2.463	0.328

LRFS, local recurrence free survival; HR, hazard ratio; CI, confidence interval; IC, induction chemotherapy; CC, concurrent chemotherapy; AC, adjuvant chemotherapy

### Risk stratification and receiver operating characteristic (ROC) curve analysis

**Fig. 3:**
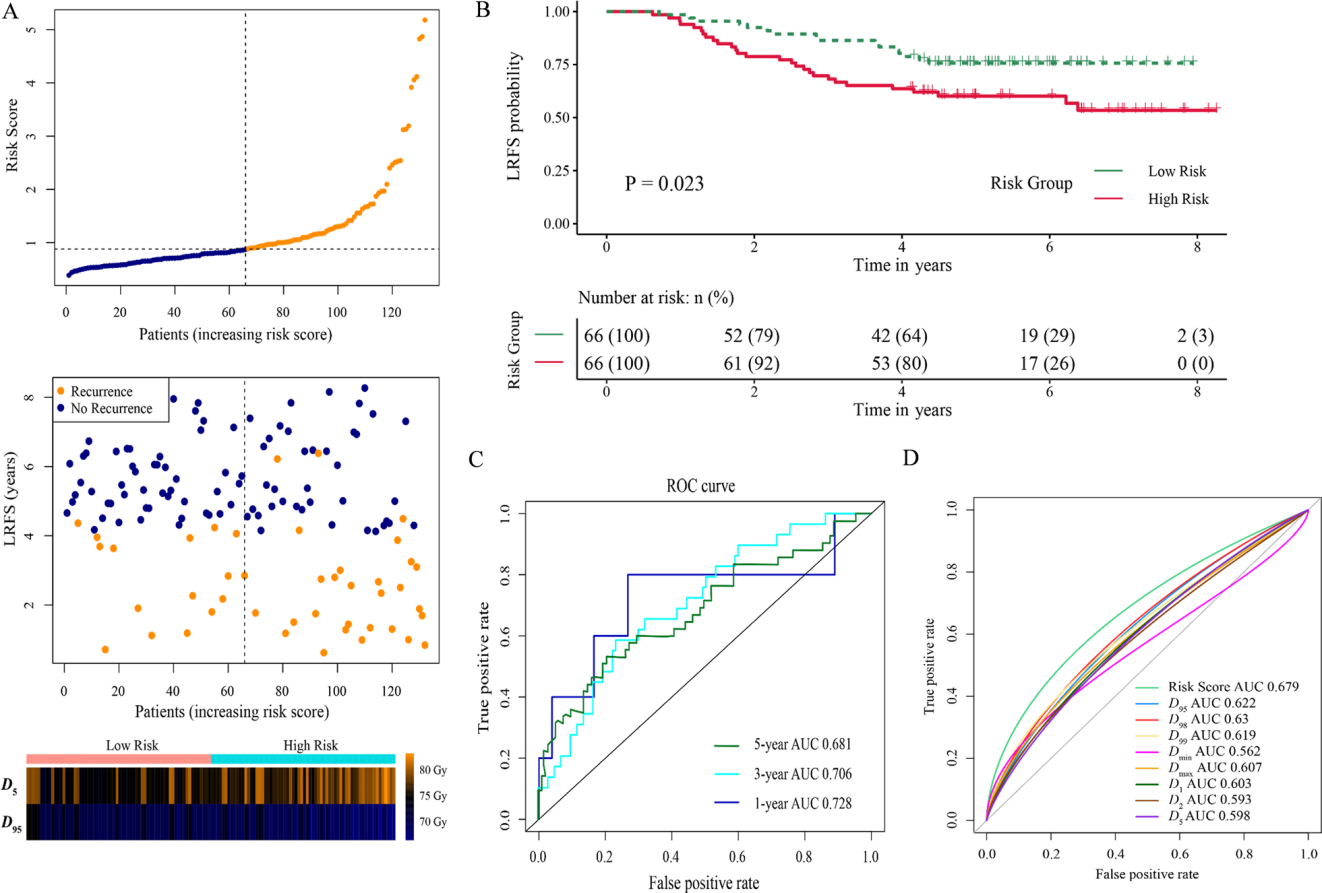
Risk score calculated by the signature of *D*_95_ and *D*_5_, Kaplan–Meier survival analysis, and time-dependent ROC curve. **a** The distribution of risk score and survival status; **b** Kaplan–Meier analysis estimated LRFS of patients according to the median value of risk score; **c** ROC curve was plotted for 1-, 3-, and 5-year LRFS; **d** Compared with other dosimetric metrics for predicting local recurrence, the risk model including *D*_5_ and *D*_95_ had the highest AUC value. LRFS, local recurrence free survival; ROC, receiver operating characteristic curve; AUC, area under curve

After obtaining the risk score for each patient according to the formula, patients were classified into low- and high-risk groups based on the median value of the score (Median value, 0.885; Range: 0.388–5.179). The distribution of the risk score along with the corresponding local recurrence data were plotted and shown in Fig. 3a. Patients with risk score ˃ 0.885 (high-risk group) tended to have a higher risk of local recurrence. The 3-year LRFS of patients in high-risk group was significantly lower than that of patients in low-risk group (66.2% vs 86.4%, *p* = 0.023) (Fig. 3b). Moreover, time-dependent ROC analysis was used to assess the predictive significance of the risk model. The area under the curve (AUC) value of ROC analysis for the prognostic signature was 0.706 and 0.681 for 3-year and 5-year LRFS, respectively (Fig. 3c). Furthermore, compared with other significant dosimetric metrics obtained from the univariate analysis, the AUC value of the risk model for predicting local recurrence was the highest (Fig. 3d).

### Comparison of HI with the risk model

**Fig. 4:**
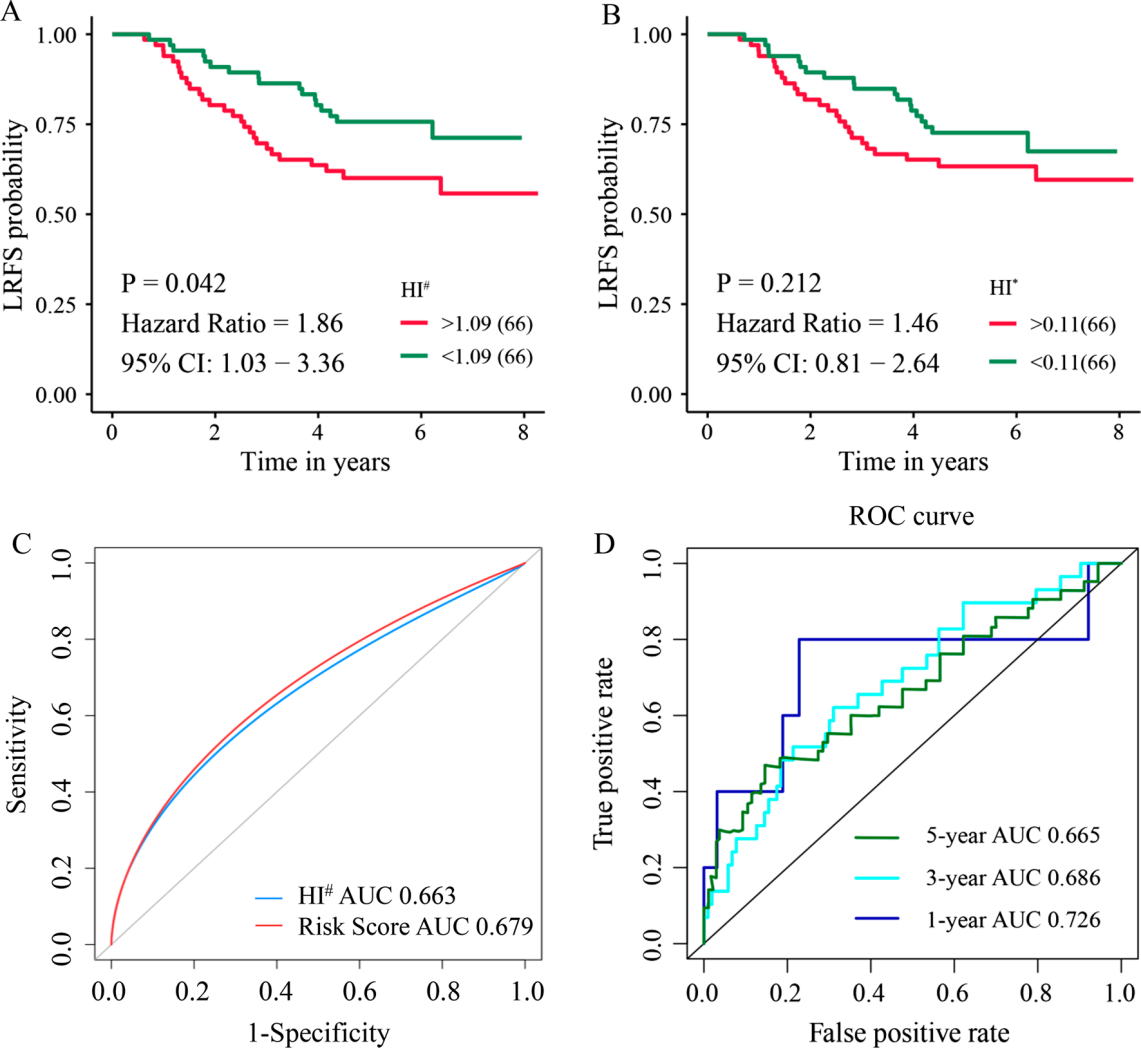
Kaplan–Meier survival analysis, and time-dependent ROC curve of HI. **a** Kaplan–Meier analysis estimated LRFS of patients according to the median value of HI^#^; **b** Kaplan–Meier analysis estimated LRFS of patients according to the median value of HI^*^; **c** Comparison of predictive power between HI^#^ and the risk model; **d** ROC curve of HI^#^ was plotted for 1-, 3-, and 5-year LRFS. HI, homogeneity index; HI^#^ was defined as *D*_5_/*D*_95_; HI^*^ was defined as (*D*_2_-*D*_98_)/*D*_50_

In order to investigate the relationship between HI and LRFS of NPC patients, univariate analysis was performed. According to the median value of HI^#^ (Median value, 1.09; Range:1.04–1.20), patients with lower HI^#^ had significantly longer LRFS compared with that with higher HI^#^ (HR 1.86; 95% CI 1.03–3.36; *p* = 0.042) (Fig. 4a). However, there was no statistical difference in LRFS between patients with lower HI^*^ (Median value, 0.11; Range:0.05–0.24) and that with higher HI^*^ (HR 1.46; 95% CI 0.81–2.64; *p* = 0.212) (Fig. 4b). Subsequently, the predictive significance of local recurrence between HI^#^ and the risk model was compared. We found that the ROC value of HI^#^ was lower than that of the risk model (AUC, 0.663 vs 0.679), although the significant difference was not reached (Fig. 4c). Furthermore, the AUC value of HI^#^ for the prognostic signature was 0.686 and 0.665 for 3-year and 5-year LRFS, respectively (Fig. 4d), which was still lower than that of the risk model (0.706 and 0.681, respectively) as analyzed before.

### Recurrent characteristics

**Fig. 5:**
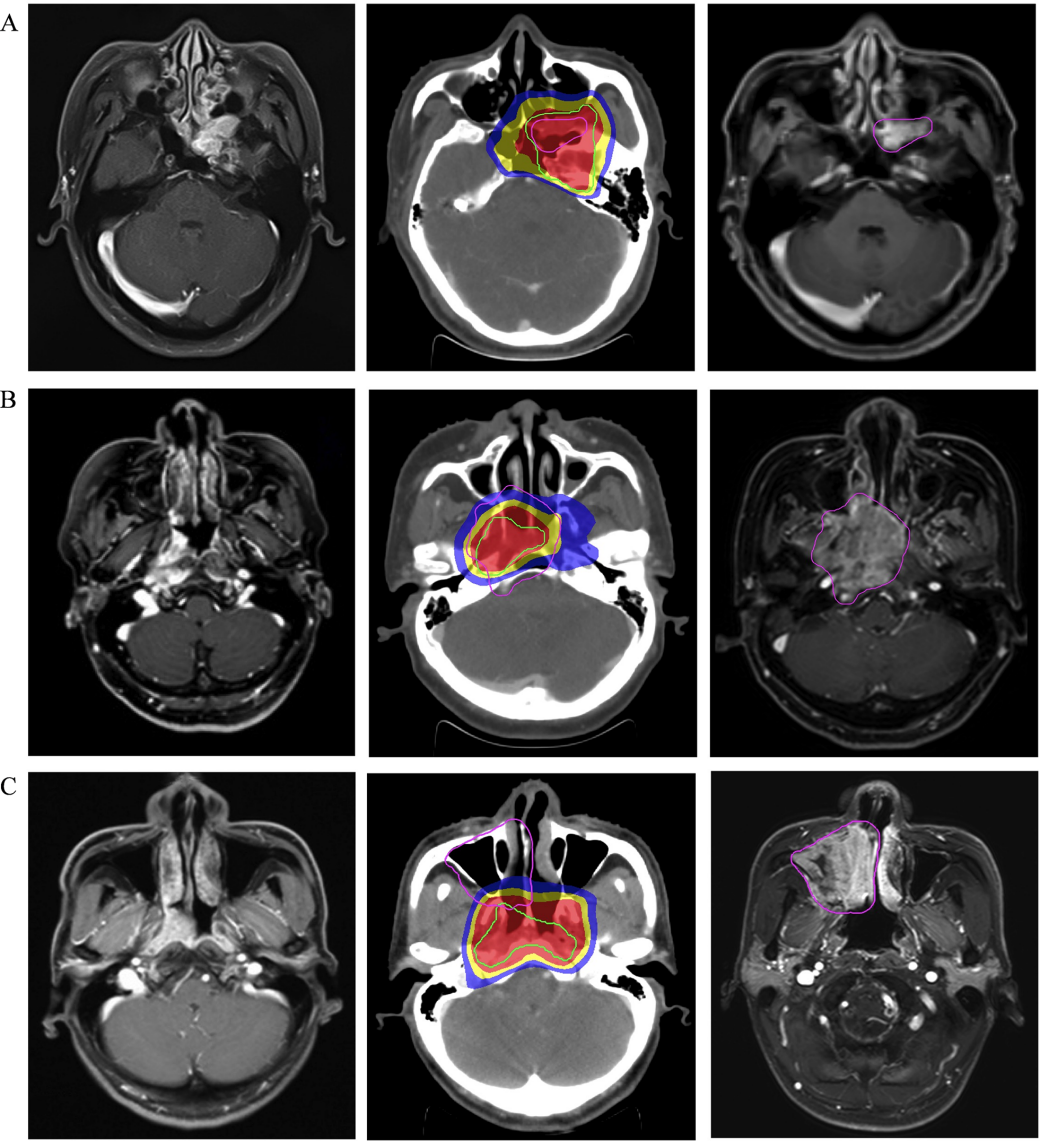
Three different types of recurrent patterns of NPC treated with IMRT. **a** in field failure; **b** marginal failure; **c** outside field failure. Left, pretreatment magnetic resonance imaging (MRI); Middle, the recurrent tumor was transferred from the MRI at the time of recurrence to the planning computed tomography (CT) to present doses to the recurrent sites; Right, MRI at the time of recurrence. The green line represented the initial gross target volume; The pink line represented the recurrent tumor volume; The red color-wash represented 70 Gy, yellow represented 66 Gy, and blue represented 60 Gy

To examine the recurrent tumor characteristics, sites of initial tumor and recurrent tumor invasion were compared. The results showed the most common recurrent site was nasopharynx cavity (n = 27, 61.4%), followed by clivus (n = 23, 52.3%) and pterygopalatine fossa (n = 18, 40.9%). Then, we compared the volume and isodose curve of recurrent tumors with those of the corresponding initial tumors. The topographic analysis showed that recurrent lesions in 30 (68.2%), 9 (20.5%) and 5 (11.3%) patients were considered as in field recurrence, marginal recurrence and outside field recurrence, respectively. Representative illustrations of the three types of recurrence were presented in Fig. 5.

## Discussion

In this study, we examined the impact of dosimetric metrics on local recurrence and analyzed recurrent characteristics of NPC patients treated with IMRT. We found that eight dosimetric metrics and HI^#^ were significantly associated with local recurrence of NPC patients while only *D*_95_ and *D*_5_ were independent prognostic factors. More importantly, a novel model constructed with these two factors could effectively predict the risk of local recurrence. Moreover, we found that in field recurrence was still the main failure pattern of NPC with IMRT, and nasopharynx cavity, clivus, and pterygopalatine fossa were the frequently recurrent sites.

IMRT was a major break-through in the treatment of NPC which dramatically enhanced the local control rate of NPC, with a 5-year LCR of 95% for T1-2 disease and 80%-88% for T3-T4 disease [21–23]. The improved LCR was associated with highly target dosimetry coverage and conformity in an IMRT planning [24, 25]. However, sometimes it is difficult to balance the conflict between the potential serious late injuries and the risk of local recurrence due to inadequate target coverage in an IMRT planning, especially in NPC patients with advanced stage [9]. Additionally, it should be also noted that quality of IMRT planning also differs due to physician’s capabilities and personal experience. The dose coverage and uniformity of the target might be inferior in certain patients, thus leading to tumor relapse. Therefore, identifying reliable dosimetric metrics associated with their treatment outcomes are significant.

In the current study, univariate and multivariate Cox regression analysis were carried out to identify important dosimetric index to predict local recurrence. First, we found that T classification was not correlated with LRFS, indicating that T classification alone had less power in dividing patients into different risk groups in IMRT era, which was similar to the result of other studies [14, 15]. However, it should be noticed that the patients in this study with T4 or bulky primary tumor received higher prescription doses (74 Gy), the conclusion might be different if these patients received lower doses (70 Gy). Although patients with advanced T classification usually had a larger tumor volume, the sophisticated IMRT technique greatly improved the dose distribution and reduced the proportion of insufficient dose-related recurrence compared to two-dimensional or three-dimensional conformal radiotherapy. However, the univariate analysis found that eight dosimetric metrics were associated with LRFS, among which *D*_5_, *D*_2_, *D*_1_, and *D*_max_ reflected the near-maximum dose of the target volume, while *D*_95_, *D*_98_, *D*_99_, and *D*_min_ reflected the near-minimum dose of the target volume to some degree, suggesting that local failure might probably associate with dose homogeneity of the target volume. Similarly, other studies also indicated that both *D*_95_ and *D*_min_ were significantly associated with local recurrence [10, 11]. Subsequently, the multivariate analysis demonstrated significant prognostic value of *D*_5_ and *D*_95_ in the LRFS of NPC patients. A cumulative risk score consisted of this two dosimetric metrics was calculated, which indicated that this two-dosimetric parameter signature independently predicted LRFS in NPC patients. And the AUC value of the ROC curve was more than 0.7 when assessing the accuracy of the signature over 3-year LRFS, suggesting that the established risk model was reliable.

In this risk model, *D*_95_ and *D*_5_ were ultimately incorporated to predict local recurrence. Although ICRU 83 reports have recommended *D*_50_ as dose-volume parameter for evaluating IMRT planning [16], it was poorly adopted in academic institutions according to a survey [26]. Furthermore, consistent with other studies, there was no correlation between *D*_50_ and local recurrence [27]. However, *D*_95_ was a commonly used dose-volume constraint in clinical practice and some clinical trials use this metric to determine prescription dose [17]. In fact, the significance of *D*_95_ in an IMRT planning was similar to *D*_98_ to some extent. As *D*_95_ increased, the near-minimum dose of the target increased, thus increasing the whole absorbed dose of the target volume, which was helpful to tumor local control. One study suggested that a *D*_min_ to the GTVnx ≥ 54.0 Gy conferred better local control in NPC patients with T3 and T4 [11], and another study also indicated that patients who received at least 66.5 Gy to primary GTV were less likely to have local failure [14]. By contrast, the significance of *D*_5_ was similar to *D*_2_. As *D*_5_ increased, the near-maximum dose of the target correspondingly raised. Hence, the dose uniformity of target was decreased, which might be harmful to tumor local control. In essence, the two dose-volume metrics of this risk model determined the shape and trend of a dose line in DVH (vertical drop or not) to some extent, reflecting a homogeneous absorbed-dose distribution in the target [16]. From this point of view, the risk model developed by this study was sensible.

Due to this risk model included the metrics of *D*_5_ and *D*_95,_ which were also the key parameters to be used for calculating the dose HI^#^. HI^#^ is also a commonly used dosimetric parameter for treatment plan reporting recommended by TG101 [19]. Hence, the univariate analysis was performed to examine the relationship between HI^#^ and local recurrence. We found that patients with higher HI^#^ had significantly shorter LRFS than that with lower HI^#^. Dose HI reflected the uniformity of the absorbed dose distribution of the target volume. As the HI^#^ increased, the “hot spot” of the target volume increased, and the “cold spot” of the target volume decreased. This meant that this IMRT planning itself was difficult, and dosimetrists might sacrifice dose coverage and uniformity of the target to reduce doses of OAR, thus increasing the risk of tumor relapse. In addition, we did not find that HI* was statistically associated with LRFS. The possible reason might be that the formula used to calculate HI* included three parameters according to ICRU 83: *D*_2_, *D*_98_, and *D*_50_. However, these parameters were not independent prognostic factors in our multivariate analysis, which was also consistent with the study of Wang et al. [27]. Therefore, our study added evidence that HI^#^ might be a more promising parameter for IMRT evaluation compared with HI^*^.

Although HI^#^ was demonstrated as an indicator for predicting local recurrence, the predictive power of HI^#^ was lower than that of the risk model according to the ROC value, especially the signature of 3-year LRFS. Therefore, compared with the HI^#^, the risk model that we established was more preferable to predict local recurrence of NPC patients.

In addition, the results of one study exploring the influence of target dosimetry on tumor recurrence in NPC differs from our results [27]. They concluded that *D*_90_ was the independent dosimetric parameter for predicting tumor recurrence and patients with *D*_90_ < 101% had higher incidence of local–regional recurrence than those with *D*_90_ > 101%. The possible reason underling the inconsistent conclusion might be that their studies focused on both local and regional lymph nodes recurrence, while ours only focused on local recurrence. Theoretically, the impact of dosimetric metrics on primary tumor is greater than on regional lymph nodes, because the latter is more influenced by anatomical change and positioning errors during radiotherapy [28–30], which might ultimately affect the analysis of dosimetric metrics on treatment outcome. From this point of view, it might be more reasonable to only focus on local recurrence when analyzing the effect of dosimetric metrics on treatment outcome, and include more factors when analyzing the factors influencing the local–regional recurrence. Nevertheless, more studies are needed to validate these conclusions.

Previous studies have showed that the local relapse of NPC mainly occurred in high dose area. In the study of Yang et al., they analyzed 212 NPC patients undergoing IMRT and found that 18 patients developed local recurrence, 15 (83.3%) of which were confirmed with in field failure [31]. Wang et al. also reported that in field failure was the main pattern associated with local–regional recurrence of NPC [15].The present study further confirmed this conclusion: in field failure was found in 68.2% recurrent patients, while marginal and outside field failure were not common. Together with those results, it was suggested that the definition and delineation of CTV currently used was large enough, with low incidence of outside field failure. Hence, further reducing CTV coverage to reduce late complications of patients is an important direction to explore in IMRT era in the future [31, 32].

This study has several limitations. Although PSM method was adopted, the selection bias was inevitable. Besides, due to the lack of more patients with local recurrence in our center, we did not have enough patients to construct another independent cohort to validate the risk model. Finally, we just focused on the dosimetric metrics of PGTVnx, other targets and OAR sparing might have some effects on outcome of patients. Given these limitations, more studies or multicenter researches are warranted.

## Conclusion

Taken together, the association between dosimetric metrics and clinical outcome was examined in this study. And, we established a novel risk model that could effectively predict the LRFS in patients with NPC, which would benefit patients who had high risk of local recurrence. Moreover, our study added more evidence for the view that *D*_95_, *D*_5,_ and HI^#^ (high hot spot and low cold spot coexist) were important metrics for dose constraint and evaluation in an IMRT planning through real-world data.

## Supplementary Information

**Additional file 1: Supplementary Table 1.** Univariate analysis of dosimetrics by Cox regression model.

**Additional file 2: Supplementary Table 2.** Two parameters from multivariate Cox regression analysis were used to develop model.

## Data Availability

The datasets used and analyzed in the study are available from the corresponding author on reasonable request.

## Abbreviations

LRFS: Local recurrence free survival;
DVH: Dose-volume histogram
HI: Homogeneity index
CR: Complete remission
AJCC: American Joint Committee on Cancer
ICRU: International Commission on Radiation Units and Measurements
TG 101: The report of AAPM Task Group 101
GTVnx: Nasopharynx gross tumor volume
GTVnd: Node gross tumor volume
CTV: Clinical target volume
PTV: Planning tumor volume
OAR: Organ at risk
SIB: Simultaneous integrated boost
IC: Induction chemotherapy
CC: Concurrent chemotherapy
AC: Adjuvant chemotherapy
RTV: Recurrent tumor volume
ROC: Receiver operating characteristic curve
