# Supplementary material for: A novel dosimetric metrics-based risk model to predict local recurrence in nasopharyngeal carcinoma patients treated with intensity-modulated radiation therapy
Source: Radiat Oncol. 2021 Sep 23;16:186. doi: 10.1186/s13014-021-01911-5 (PMC8461883; doi:10.1186/s13014-021-01911-5)
Supplement: Supplementary file 1 — Additional file 1: Supplementary Table 1. Univariate analysis of dosimetrics by Cox regression model. [file 13014_2021_1911_MOESM1_ESM.docx]

**Supplementary table 1** Univariate analysis of dosimetric metrics by Cox regression model

| Variable | HR | 95% CI | | *P* value |
| --- | --- | --- | --- | --- |
|  |  | lower | upper |  |
| *D_95_* | 0.99763 | 0.99564 | 0.99962 | **0.01971** |
| *D_98_* | 0.99780 | 0.99644 | 0.99915 | **0.00150** |
| *D_99_* | 0.99840 | 0.99740 | 0.99940 | **0.00179** |
| *D_90_* | 0.99916 | 0.99728 | 1.00104 | 0.38145 |
| *D_min_* | 0.99953 | 0.99909 | 0.99997 | **0.03771** |
| *D_85_* | 0.99974 | 0.99800 | 1.00148 | 0.77341 |
| *D_80_* | 0.99997 | 0.99831 | 1.00163 | 0.97579 |
| *D_75_* | 1.00017 | 0.99859 | 1.00176 | 0.82817 |
| *D_70_* | 1.00031 | 0.99878 | 1.00183 | 0.69062 |
| *D_65_* | 1.00040 | 0.99892 | 1.00188 | 0.59192 |
| *D_60_* | 1.00047 | 0.99903 | 1.00192 | 0.51703 |
| *D_55_* | 1.00056 | 0.99914 | 1.00198 | 0.43468 |
| *D_ave_* | 1.00058 | 0.99911 | 1.00205 | 0.43897 |
| *D_50_* | 1.00064 | 0.99925 | 1.00203 | 0.36371 |
| *D_45_* | 1.00070 | 0.99934 | 1.00206 | 0.31081 |
| *D_40_* | 1.00077 | 0.99943 | 1.00210 | 0.25932 |
| *D_35_* | 1.00083 | 0.99952 | 1.00215 | 0.21177 |
| *D_30_* | 1.00089 | 0.99960 | 1.00218 | 0.17385 |
| *D_25_* | 1.00094 | 0.99967 | 1.00221 | 0.14548 |
| *D_20_* | 1.00100 | 0.99976 | 1.00224 | 0.11347 |
| *D_15_* | 1.00106 | 0.99984 | 1.00227 | 0.0866 |
| *D_10_* | 1.00112 | 0.99993 | 1.00230 | 0.06377 |
| *D_max_* | 1.00115 | 1.00018 | 1.00213 | **0.01984** |
| *D_2_* | 1.00115 | 1.00006 | 1.00225 | **0.03803** |
| *D_1_* | 1.00116 | 1.00009 | 1.00223 | **0.03232** |
| *D_5_* | 1.00117 | 1.00003 | 1.00231 | **0.04409** |

Notes: *D*_x_ was defined as the minimum absorbed dose that covers x% of the volume of the target; *D*_ave_ represented the average dose of the target. *D*_min_ represented the minimum dose of the target. *D*_max_ represented the maximum dose of the target.

Abbreviations: HR, hazard ratio; CI, confidence interval.
