# Supplementary material for: A novel dosimetric metrics-based risk model to predict local recurrence in nasopharyngeal carcinoma patients treated with intensity-modulated radiation therapy
Source: Radiat Oncol. 2021 Sep 23;16:186. doi: 10.1186/s13014-021-01911-5 (PMC8461883; doi:10.1186/s13014-021-01911-5)
Supplement: Supplementary file 2 — Additional file 2: Supplementary Table 2. Two parameters from multivariate Cox regression analysis were used to develop model. [file 13014_2021_1911_MOESM2_ESM.docx]

**Supplementary table 2** Two parameters from multivariate Cox regression analysis were used to develop model

| Variable | HR | β coefficient | 95% CI of HR | | *P* value |
| --- | --- | --- | --- | --- | --- |
|  |  |  | lower | upper |  |
| *D_5_* | 1.00189 | 0.0019 | 1.00128 | 1.00249 | 0.002 |
| *D_95_* | 0.99702 | -0.0030 | 0.99612 | 0.99792 | < 0.001 |

Notes: *D*_x_ was defined as the minimum absorbed dose that covers x% of the volume of the target.

Abbreviations: HR, hazard ratio; CI, confidence interval.
